# Measurement of single kidney glomerular filtration rate in dogs using dynamic contrast-enhanced magnetic resonance imaging and the Rutland-Patlak plot technique

**DOI:** 10.1186/s13028-018-0423-3

**Published:** 2018-11-06

**Authors:** Jan-Niklas Mehl, Matthias Lüpke, Ann-Cathrin Brenner, Peter Dziallas, Patrick Wefstaedt, Hermann Seifert

**Affiliations:** 1 Institute for General Radiology and Medical Physics, University of Veterinary Medicine Hannover, Foundation, Bischofsholer Damm 15, 30173 Hannover, Germany; 2 Small Animal Clinic, University of Veterinary Medicine Hannover, Foundation, Bünteweg 9, 30559 Hannover, Germany

**Keywords:** Dog, Dynamic contrast-enhance MRI, Glomerular filtration rate, Kidney, Renal function, Rutland-Patlak plot

## Abstract

**Background:**

Nephropathies are among the most common diseases in dogs. Regular examination of the kidney function plays an important role for an adequate treatment scheme. The determination of the glomerular filtration rate (GFR) is seen as the gold standard in assessing the kidney status. Most of the tests have the disadvantage that only the complete glomerular filtration rate of both kidneys can be assessed and not the single kidney glomerular filtration rate. Imaging examination techniques like dynamic contrast-enhanced magnetic resonance imaging have the potential to evaluate the single kidney GFR. There are studies in human medicine describing the determination of the single kidney GFR using this technique. To our knowledge there are no such studies for dogs.

**Results:**

An exponential fit was found to describe the functional interrelation between signal intensity and contrast medium concentrations. The changes of contrast medium concentrations during the contrast medium bolus propagation were calculated. The extreme values of contrast medium concentrations in the kidneys were reached at nearly the same time in every individual dog (1st maximum aorta 8.5 s, 1st maximum in both kidneys after about 14.5 s; maximum concentration values varied between 17 and 125 µmol/mL in the aorta and between 4 and 15 µmol/mL in the kidneys). The glomerular filtration rate was calculated from the concentration changes of the contrast medium using a modified Rutland-Patlak plot technique. The GFR was 12.7 ± 2.9 mL/min m^2^ BS for the left kidney and 12.0 ± 2.2 mL/min/m^2^ BS for the right kidney. The mean values of the coefficient of determination of the regression lines were averagely 0.91 ± 0.08.

**Conclusions:**

The propagation of contrast medium bolus could be depicted well. The contrast medium proceeded in a similar manner for every individual dog. Additionally, the evaluation of the single kidney function of the individual dogs is possible with this method. A standardized examination procedure would be recommended in order to minimize influencing parameters.

**Electronic supplementary material:**

The online version of this article (10.1186/s13028-018-0423-3) contains supplementary material, which is available to authorized users.

## Background

In companion animal medicine, the importance of canine nephropathies should not be underestimated. The prevalence of chronic kidney disease (CKD) have been assessed to be up to 3.74% [1–4]. The progressive course of this disease requires a changing treatment scheme. Therefore, periodic control examinations are recommended for an adequate treatment [5]. Endogenous creatinine and blood urea nitrogen are used most frequently in clinical practise for evaluating the renal function but these are neither sensitive enough to reveal subclinical or border-line renal failure [6, 7], nor are they suitable for evaluating single kidney function [8, 9]. The measurement of the glomerular filtration rate (GFR) is classified as being the best single test for assessing kidney function [10]. Despite this, this tool is seldom used in veterinary medicine because of its high cost and effort [6]. It might also be a useful screening method for early monitoring the kidney function of dog breeds that are predisposed for nephropathies [6].

Inulin-clearance is reputed to be the gold standard for GFR measurement. However, this examination can only be performed under elaborate clinical conditions [11]. Creatinine [6, 7, 12, 13] and Gadolinium-1,4,7,10-tetraazacyclododecane-1,4,7,10-tetraacetic acid (Gd-DOTA) [8, 14] are seen as eligible markers for measuring GFR in humans and rats.

As stated in numerous studies in human medicine, functional magnetic resonance imaging (fMRI) is used to assess the GFR. The main advantage is the absence of ionising radiation. This fact should not be underestimated regarding repeated evaluations of the kidney status.

Nevertheless, MRI devices are still not widely distributed in veterinary clinics and high costs for this kind of examination have to be mentioned [15]. Additionally, limitations of renal fMRI examinations contribute to the lack of agreement on the quantification of the concentration of the contrast medium depending on signal intensity (SI) as well as a standardised protocol for the examination and analysis [16].

There is no consensus on a suitable model for the calculation of GFR either [17, 18]. Previous studies introduced or developed different models for GFR measurements including various amounts of compartments [18–23]. GFR was calculated in our study using a modified Rutland-Patlak plot (RPP) model. The model was used the first time by Hackstein et al. [19] to determine the GFR via fMRI measurements and describes a graphical solution of a simplified two-compartment model [19]. The main advantage of this model is its simplicity because it is just a two-compartment model and no other physiological parameters are needed for calculating the GFR [21].

The fMRI-data were analysed to answer the following questions: (1) Is it possible to evaluate the single-kidney functions in dogs using fMRI-bolus-tracking? and (2) How much influence do different evaluation parameters have on the calculation of the modified RPP?

## Methods

### Dogs

Eight healthy Beagle dogs (four males and four females) were included. They were kept as experimental animals at the Clinic for Small Animals at the University of Veterinary Medicine Hannover, Foundation, Germany. All procedures were approved by the animal welfare officer of the University of Veterinary Medicine Hannover, Foundation and the Lower Saxony State Office for Consumer Protection and Food Safety, Oldenburg, Germany (TV-No. 33.9-42502-04-08/1600).

The age of the dogs ranged from 4 to 11 years with a mean age of 8.5 years and a standard deviation of 2.9 years. The body weight (BW) of the dogs ranged from 14 kg to 22 kg with a mean BW of 17.5 kg and a standard deviation of 2.7 kg.

### GFR measurement by the clearance of exogenously administered creatinine

To evaluate the kidneys’ health status, the dogs’ GFR was measured by determining the modified plasma-clearance of exogenously administered creatinine according to a test that has been evaluated previously [24, 25].

This method is accepted as a simple and accurate method [6, 24, 25] that can be reliably performed in clinical daily routine without special elaborate examination methods [7, 12, 13].

An exact calculated amount of 5% creatinine solution (LABOKLIN GmbH & Co.KG, 97688 Bad Kissingen, Germany) depending on the body surface (BS) of the dog was injected subcutaneously in every dog (2 g creatinine/m^2^ BS). BS was calculated with the help of the BW.

$$BS [{\text{m}}^{2} ] = 0.1 \cdot BW \, [{\text{kg}}]^{0.667}$$

According to the test specifications four blood samples were taken per dog. They were sent to an external laboratory (LABOKLIN GmbH & Co.KG) and analysed there.

### Serial dilution

For analysing the fMRI measurements in vivo a functional correlation between SI and concentration of contrast medium had to be established. Therefore, a serial dilution of the contrast medium was made. 25 test tubes were filled with 0.9% NaCl and the contrast medium in concentrations from 0 to 80 mmol/L. All tubes were put into a water quench which was heated up to 38 °C and examined in MRI with the same settings as the fMRI measurements for the dogs (see Table 1—bolus track).

**Table 1:** MR sequence parameters

Parameter	Bolus track	Anatomical sequence
Sequence	T1-FFE = fast field echo = gradient echo sequence	T2 W-TSE_HR = Turbo-Spin-Echo
Repetition time (ms)	4.2628	1510.694
Echo time (ms)	1.281	100
Flip angle	40°	90°
Voxel size (mm)	1.74 × 1.74 × 45	0.73 × 0.73 × 5
Time between two slices (s)	0.58	
Slice thickness (mm)	45	5
Slice orientation	Dorsal	Transversal/dorsal

All MRI examinations were performed with a Philips Achieva 3 Tesla scanner.

Due to turbulence in the water, this measurement was read 4 times at intervals of 14, 106 and 127 min. The mean SIs of the different contrast medium concentrations were determined. The functional correlation between SI and concentration of contrast medium was calculated from 0 to 15 mmol/L (SI 0–1200) using the software Origin Pro^®^ (OriginLab Corporation, Massachusetts, USA). As fit-functions a third-degree polynomial and an ascending exponential function were chosen.

### MRI examination of the dogs

For preparing the anaesthesia and injecting the contrast medium all dogs were given a vein catheter either in the cephalic vein or the saphenous vein. An extension line type Heidelberger was attached to the vein catheter in order to administer the contrast medium manually during the examinations. The anaesthesia of all dogs was started with an injection of levomethadon [0.2 mg/kg intravenously (i.v.)], diazepam (1 mL/10 kg i.v.) and propofol (4–6 mg/kg i.v.). Hereafter, the dogs were intubated, and the anaesthesia was continued by inhalant anaesthesia (1–1.2% end-tidal expired isoflurane).

All dogs were in a supine position for the MRI-examinations and a body coil was used. The pre-settings for the fMRI sequence (bolus track) were chosen according to Table 1. The images of the sequence are created in a so called subtraction procedure. This means that the SIs of the third image in the sequence are taken as reference values and subtracted from all subsequent images. Every fMRI examination took about 15 min (see Additional file 1).

### Bolus track

The contrast medium (Dotarem 0.5 mmol/mL; Querbet, 95943 RoissyCdGCedex, France) was injected as bolus at a dose of 0.1 mmol/kg (0.2 mL/kg). To insert the complete amount of contrast medium first 6 mL of 0.9% NaCl were poured into the extension line type Heidelberger. After that the contrast medium was injected and finally the extension line was rinsed with 15 mL of 0.9% NaCl.

### Image analysis

**Fig. 1:**
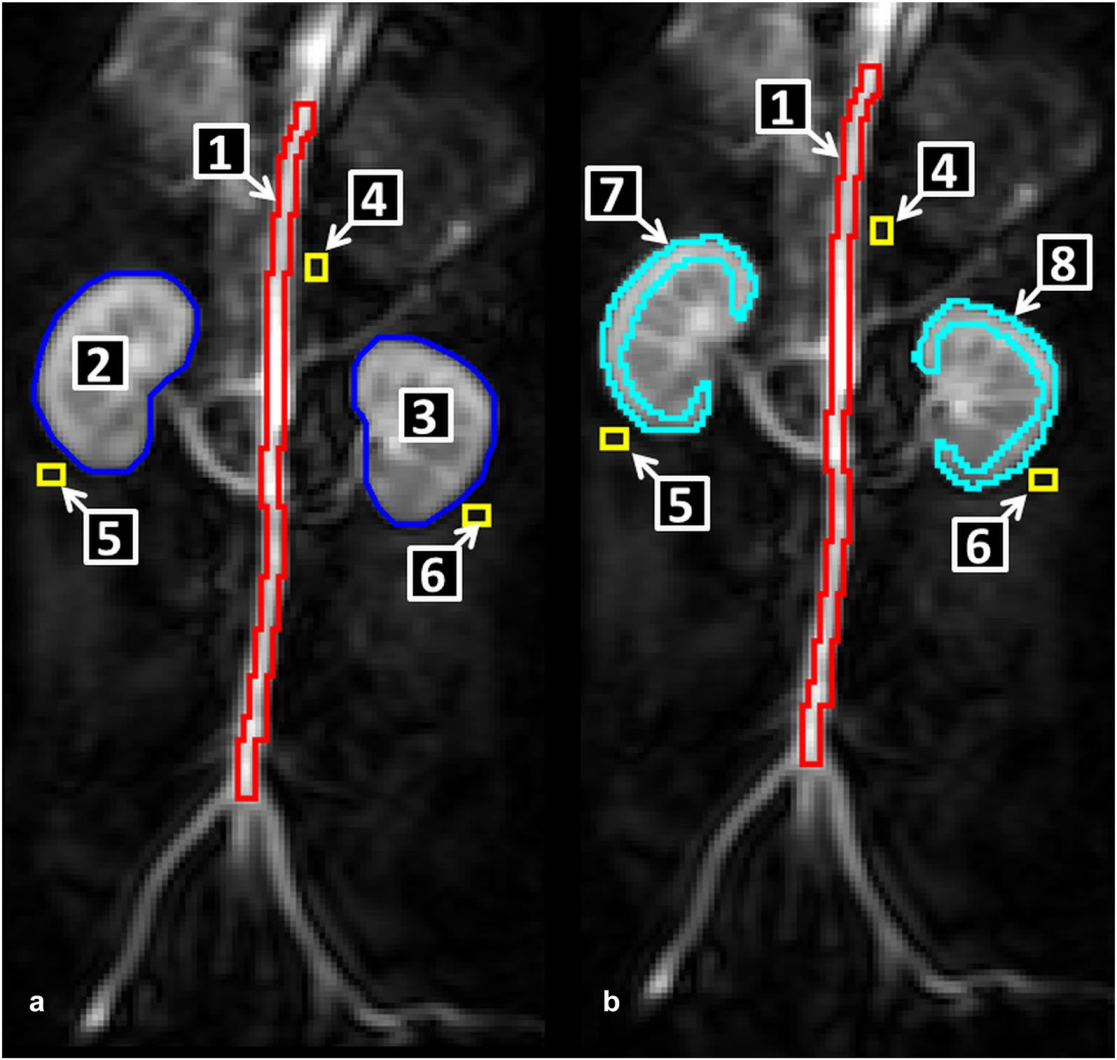
Localisation of different ROIs for analysing the Bolus track sequence. The different numbers represent: 1 ROI_aorta all_ (red), 2 ROI_right kidney_ (blue), 3 ROI_left kidney_ (blue), 4 ROI_correction aorta_ (yellow), 5 ROI_correction right kidney_ (yellow), 6 ROI_correction left kidney_ (yellow), 7 ROI_right cortex_ (cyan) and 8 ROI_left cortex_ (cyan)

In order to measure the SI changes of the functional MR-images, the sequences were loaded in the computer software ImageJ^®^. After that, manually drawn regions of interest (ROIs) were created once for all slices. The ROIs were put over both kidneys and cortices. Furthermore, a ROI was drawn over the aorta representing the vascular space. All ROIs were created within the boundaries of the tissues. Additionally, small rectangular ROIs were drawn closely to the left and right kidney as the aorta to establish a correction factor (Fig. 1). They were needed to calculate the concentrations of contrast medium in the organs.

First of all we compared the progression of SI changes in all the different ROIs of all dogs. The mean values of the SI-changes were measured until the end of MRI-examination and transferred to Excel^®^ (Microsoft Corporation).

To convert the SIs of the different ROIs to concentrations of contrast medium in the ROIs the functional correlation of the serial dilution was used.

The slice thickness of the fMRI examinations was thicker than the volume of the relevant organs.

Therefore the complete kidney could be implied for the calculation of the GFR and not just a small part of the renal parenchym. Additionally an enlarged slice thickness leads to an improved signal to noise ratio.

Thus, SI in the voxels of the measured ROIs contained SI contributions of the organs and the residual tissues.

Therefore, the distortion caused by the residual tissue had to be corrected. In order to calculate the volume of the organs in the ROIs, the diameters of aorta, kidneys and cortices were measured in the T2-weighted images (Table 1). After that, the volume of the residual tissue was computed by subtracting the volumes of the organs. The real concentration c_tissue_ in the tissues could be calculated according to the following formula.

$$c_{tissue} = c_{Voxel} \times \frac{{V_{Voxel} }}{{V_{tissue} }} - c_{residual\,tissue} \times \frac{{V_{residual\,tissue} }}{{V_{tissue} }}$$

c_Voxel_ is the concentration of contrast medium in the complete voxel, V_Voxel_ is the volume of the voxel, V_tissue_ is the measured volume of the tissue in the voxel, c_residual tissue_ is the concentration of the contrast medium in the residual space of the voxel, and V_residual tissue_ is the volume of the residual tissue.

To eliminate the artefacts which were mainly caused by breathing movement a Savitzky–Golay filter [26] was used. The best results were found when the filter was put over 15 pictures (8.7 s).

For all dogs, the temporal changes of contrast medium concentrations were calculated and compared. Starting point for integration (t = 0 s) was the last image before a SI ascent caused by the arrival of the contrast bolus in the ROI_aorta_ could be measured.

### Rutland-Patlak plot

In order to calculate the single kidney GFR a modified Rutland-Patlak plot (RPP) was computed using the previously calculated time-dependent changes of the contrast medium concentrations in the affected organs. As stated previously [20] the RPP had to be modified due to the delayed propagation of the contrast medium in the renal vessels compared to the aorta. To fulfil all requirements of the RPP-model [19], the course of the concentrations in the kidneys was shifted by a time span (Δt). Thus, the RPP-formula was modified by Δt. The final RPP-formula sets up a straight line equation. The y-value is plotted against the x-value to calculate the gradient p.V_vas_ graphically. This enables the single kidney GFR to be calculated.

$$\underbrace {{\frac{{c_{kidney} \left( {t + \Delta t} \right) \cdot V_{kidney} }}{{c_{aorta} \left( t \right)}}}}_{\text{y}} = V_{vas} + p \cdot V_{vas} \cdot \underbrace {{\frac{{\mathop \smallint \nolimits_{0}^{t} c_{aorta} \left( {t^{\prime}} \right) dt^{\prime}}}{{c_{aorta} \left( t \right)}}}}_{\text{x}} \Leftrightarrow y = b + m \cdot x$$

c_kidney_ is the concentration of contrast medium in the kidney, V_kidney_ is the volume of the kidney and c_aorta_ is the concentration in the aorta. V_vas_ is the volume of the vascular space and p is the constant of proportionality.

The time span between the first concentration maxima in the ROI_aorta_ and ROI_kidney_ was taken as Δt.

Due to the heterogeneous propagation of the contrast bolus in the different dogs the starting point for integration was set to the point of time when the second maximum concentration was reached in the aorta. The time interval of the RPPs was 60 s. The starting point also marked the first pair of the x-value and y-value that was plotted in the RPPs.

In order to calculate the glomerular filtration rate of a single kidney the gradient had to be multiplied by 60 and divided by the BS in order to state the results in mL/min/m^2^ BS. To calculate the GFR of the plasma the results were multiplied by the factor [1 − hematocrit (hct)]. As the hct had not been measured during MRI-examination and all dogs were seen as clinically healthy, a typical hct of 0.47 was assumed according to the study of Bourgès-Abella who tried to establish reference values for Beagles, which were held under laboratory conditions [27].

**Fig. 2:**
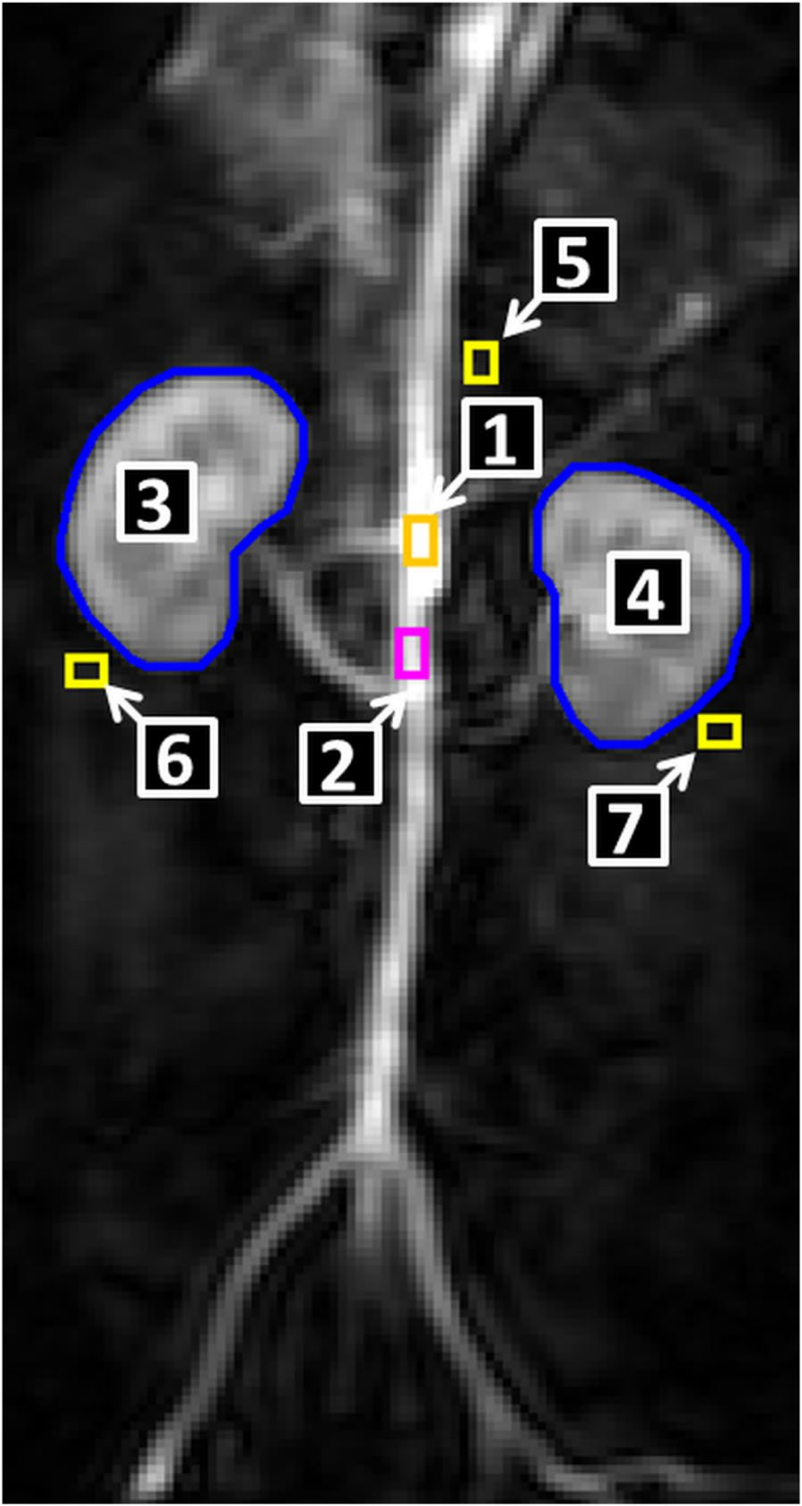
Different ROIs drawn over the aorta to measure the influence of ROI placement. ROI_highest SI_ (1; orange) ROI_a.renalis dexter_ (2; magenta); ROI_kidneys_ (3, 4; blue); ROI_correction aorta_ (yellow; 5), ROI_correction right kidney_ (yellow, 6), ROI_correction left kidney_ (yellow, 7)

Additionally, the influence of different sizes of ROI_aorta_ on the calculation of the GFR was determined. As displayed in Fig. 2 two more ROIs were drawn over the aorta in order to measure the influence of size and localisation of the ROIs in the vascular space. One rectangular ROI was drawn above the bifurcation of the arteria renalis dexter (ROI_a.renalis dexter_) having the dimensions 3 voxels × 5 voxels; a second rectangular ROI (ROI_highest SI_) of the same size was drawn over the point where the highest value of SI could be measured. A RPP was computed for these different ROIs.

### Statistical analysis

For statistical analysis the computer software OriginPro^®^ (OriginLab Corporation, Massachusetts, USA) was used. The different RPPs were compared using box-plots. Additionally, a t-test of paired samples was performed to compare the left and right kidney function.

## Results

### Reference method

All concentrations of the dogs’ endogenous serum-creatinine values were within the limits of the reference range as well as the results for the calculation of the “modified plasma-creatinine-clearance”. In summary, all eight tested dogs could be judged healthy with regard to the kidney function according to the reference method.

### Serial dilution

**Fig. 3:**
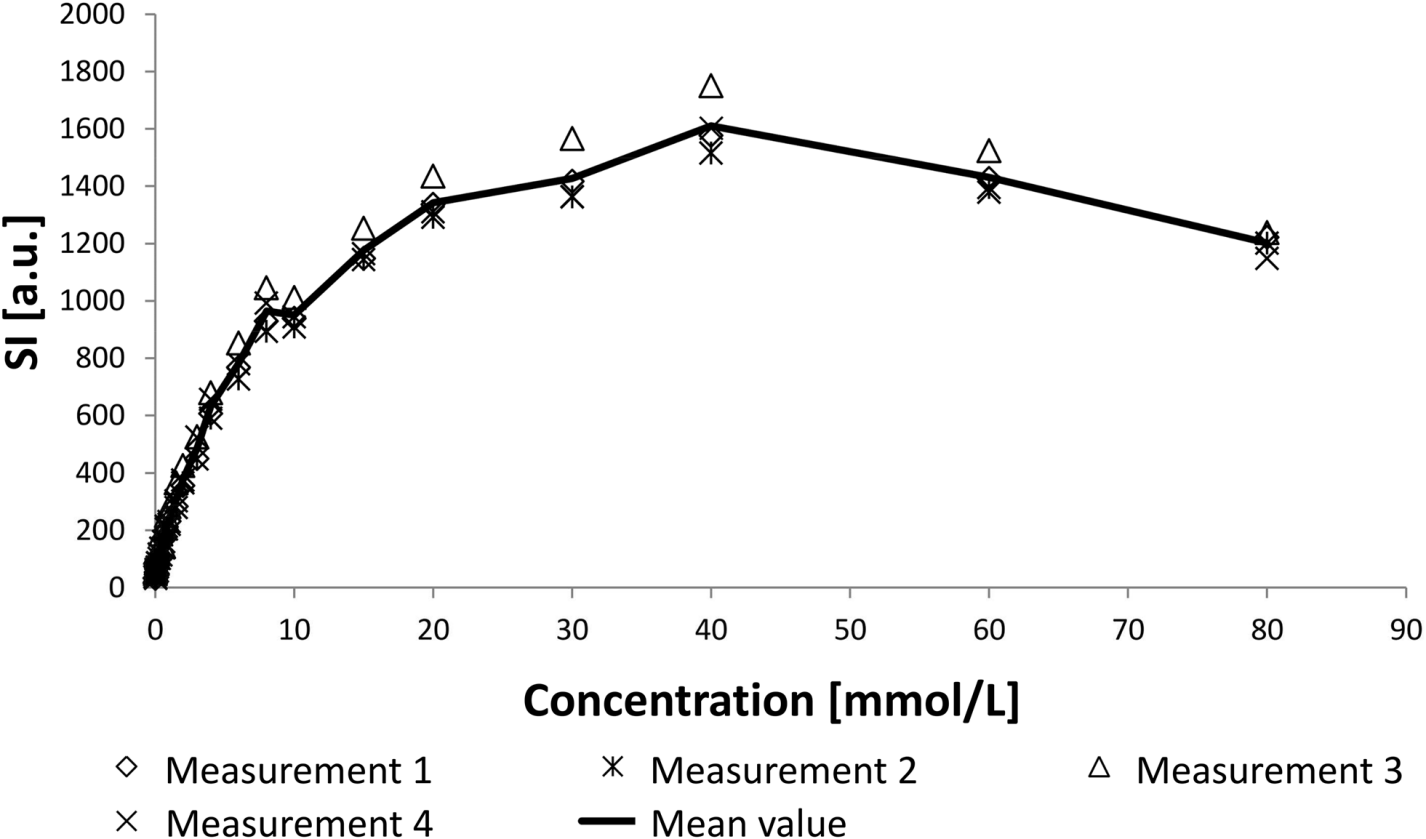
SI depending on the concentration [mmol/L] of contrast medium. After a steep incline at low concentrations a plateau was reached at concentrations of about 40 mmol/L. The more the concentration increased a diminished SI could be observed due to T_2_*-effects

The measured values and the mean value of all four measurements can be seen in Fig. 3. The results of all four single measurements are displayed by different symbols and the mean value by a continuous line. At first SI rose with increasing concentration of contrast medium, then reached a maximum value and decreased again.

As demonstrated in Fig. 3 the correlation of SI and the concentration of the contrast medium was not linear. Although the SI exceeded 1200 for three dogs for a very short time, the fit function between SI and contrast medium was just computed in this range. The fitting in this range showed the best results for the lower concentrations and a pretty good approximation for the higher values.

**Fig. 4:**
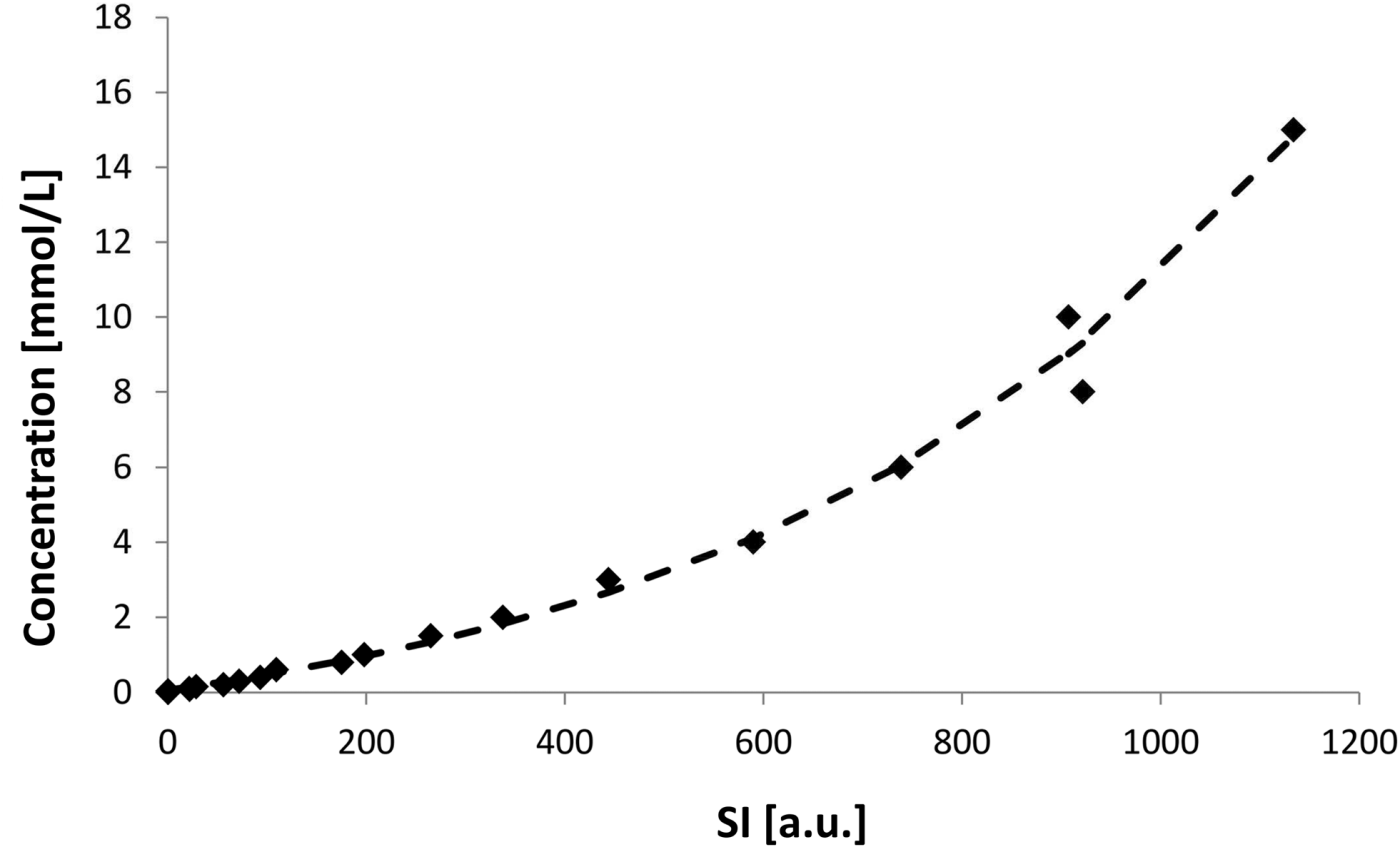
Exponential fit curve for concentration of contrast medium (mmol/L) depending on the SI from 0 to 15 (mmol/L). The symbols represent the mean values and the dotted line the fit curve

The best results were found for an exponential ascent and a third degree polynomial. Comparing these two functions using the Akaike information criteria (AIC) [28] showed that the exponential ascent was with higher probability correct (4.8 times higher). The exponential fit curve is shown in Fig. 4 and described by the following equation:

$$c_{Gadolinium} \left[ {\text{mmol/L}} \right] = a + b \cdot e^{{x \cdot S\left[ {a.u.} \right]}}$$

“c_Gadolinium_” is the concentration of Gadolinium-DOTA, “a” = − 1.933, “b” = 1.996, “x” = 0.001876 and “S” is the SI.

### Image analysis

The time course and spatial extension of contrast medium could be seen very clearly through the whole bolus track sequence of every dog. For every ROI an unambiguous increase and decrease in SI could be measured.

**Fig. 5:**
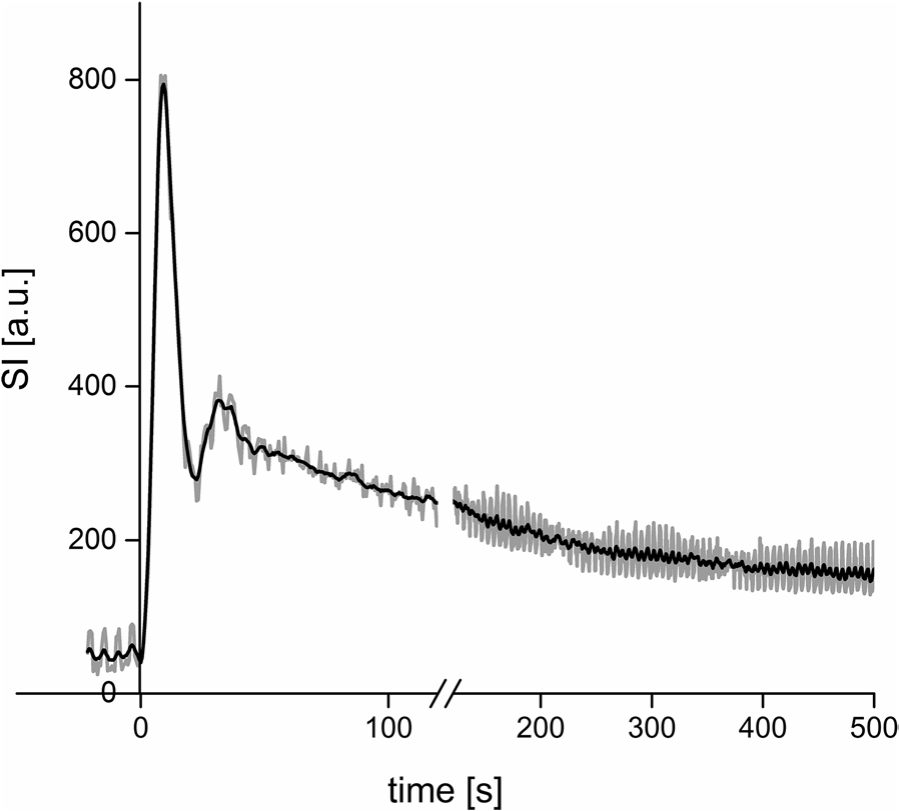
SI-changes during the propagation of the contrast bolus of one dog. The use of the Savitzky–Golay filter helped to eliminate artefacts mainly caused by breathing movement. Artefacts caused by breathing movement were nearly completely eliminated. The extreme values are displayed satisfactorily according to the point of time and the height of SI. The grey line represents the raw data whereas the continuous line demonstrates the smoothed curve. After 120 s the graphical representation of the timeline was shortened

With the determined equation all values for SI could be converted to definite concentrations in every ROI. As demonstrated in Fig. 5 smoothing the curves in the graphs helped to identify and interpret the extreme values of concentration of contrast medium and curve progression. The filter was set over 15 pictures all the time.

**Fig. 6:**
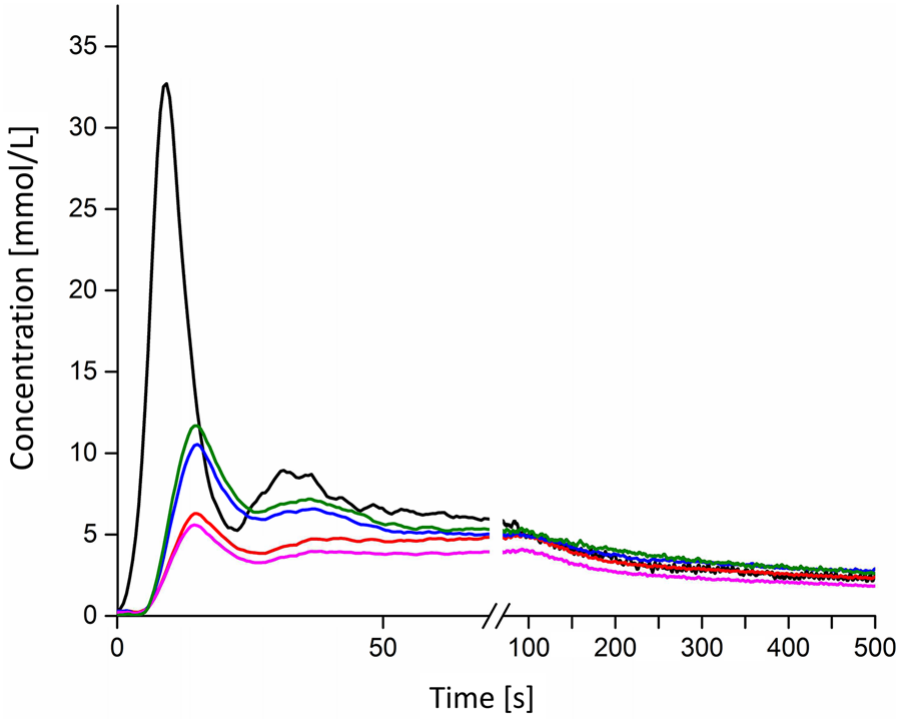
Representative propagation of the bolus track during the examination of one dog. The blackline represents the propagation of the concentrations of contrast medium through the aorta, the red line shows the concentration of contrast medium in the left kidney, the magenta line the right kidney, the blue line the left cortex and the green line the right cortex. The second maximum could be noticed mainly in the aorta and not clearly be identified in the ROIs placed over the kidneys and cortices

After corrections concerning the residual tissue had been performed the curve progression showed a pretty similar shape for each dog. As displayed in Fig. 6 the characteristic propagation of the contrast medium’s concentration in ROI_aorta all_ culminated in a first maximum value after the first increase. Afterwards, concentration decreased until a characteristically minimum value. Hereafter, the concentration of contrast medium increased again until a second lower maximum and after that it decreased continuously again. The second maximum was influenced mainly by the bolus of contrast medium that ran a second time through the aorta.

A similar curve propagation could also be monitored for the ROIs of both kidneys and cortexes: in comparison to ROI_aorta all_ a timely individually slightly delayed increase in SI could be monitored for all eight dogs.

**Fig. 7:**
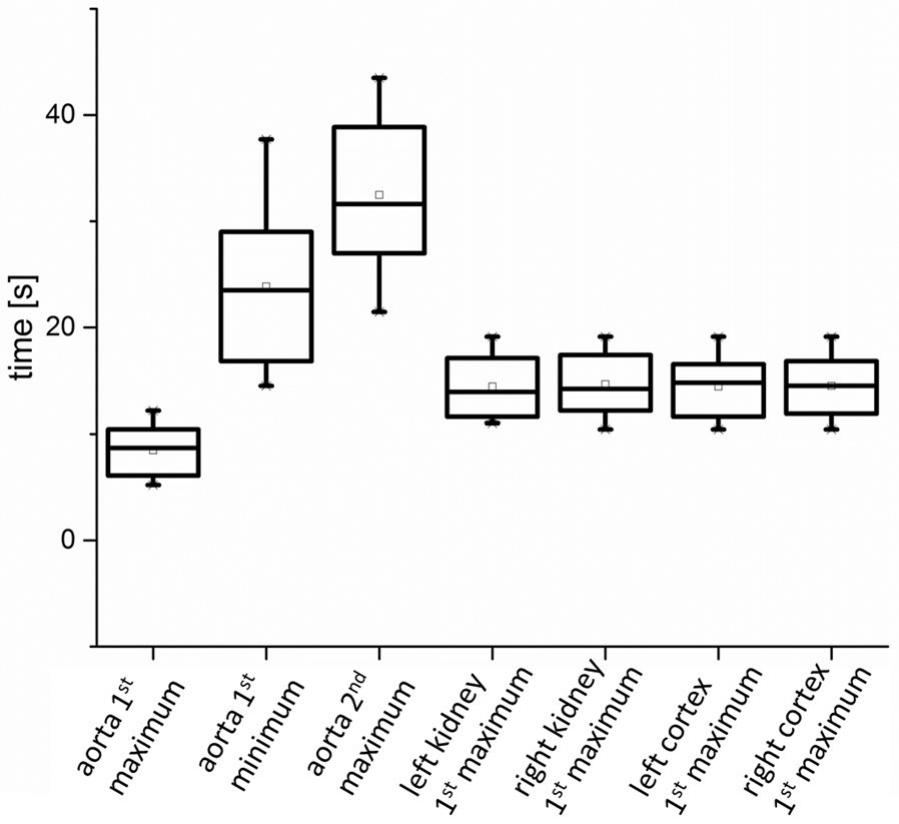
Comparison of points of time when extreme values were reached during the bolus track of the different dogs. The comparison of the left and right side demonstrated that the first maximum was reached nearly at the same time both for the kidneys and for the cortices and as well for the kidneys and cortices in general

**Fig. 8:**
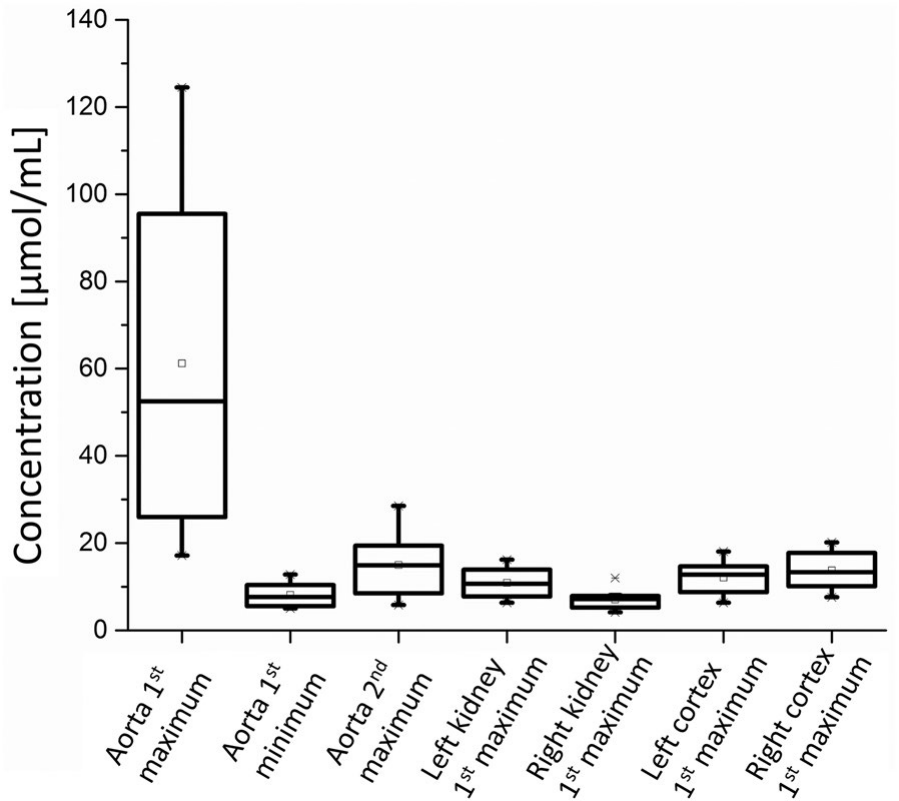
Concentration of contrast medium obtained at the extreme values during the bolus track. The measured concentrations of the different dogs for ROI_aorta all_ varied greatly

Although the curve propagation of all dogs was of a similar nature the extreme values were reached at different point of times. Additionally, the concentration values of contrast medium varied considerably. All points of time when the extreme values in the different ROIs were reached are listed in the boxplot in Fig. 7 and the associated concentrations in Fig. 8.

The first peak of ROI_aorta all_ occurred on average after 8.5 s, the first minimum after 23.9 s and the second maximum after 32.3 s. The maximum in all ROIs of cortexes and kidneys was reached after 14.5 s. Comparing the point of times of every single dog’s left and right first maximum resulted in a margin of 0.2 s for the ROIs_kidney_ and 0.1 s for the ROIs_cortex_.

The comparison of concentration of contrast medium (Fig. 8) showed a wide span for ROI_aorta all_ from 17 µmol/mL to 125 µmol/mL. The concentration in the kidneys varied from 4 µmol/mL to 16 µmol/mL and in the cortexes from 6 µmol/mL to 20 µmol/mL. The differences between the left and right side were 3.8 µmol/mL for the kidneys and 1.7 µmol/mL for the cortices.

### Rutland-Patlak plot

**Fig. 9:**
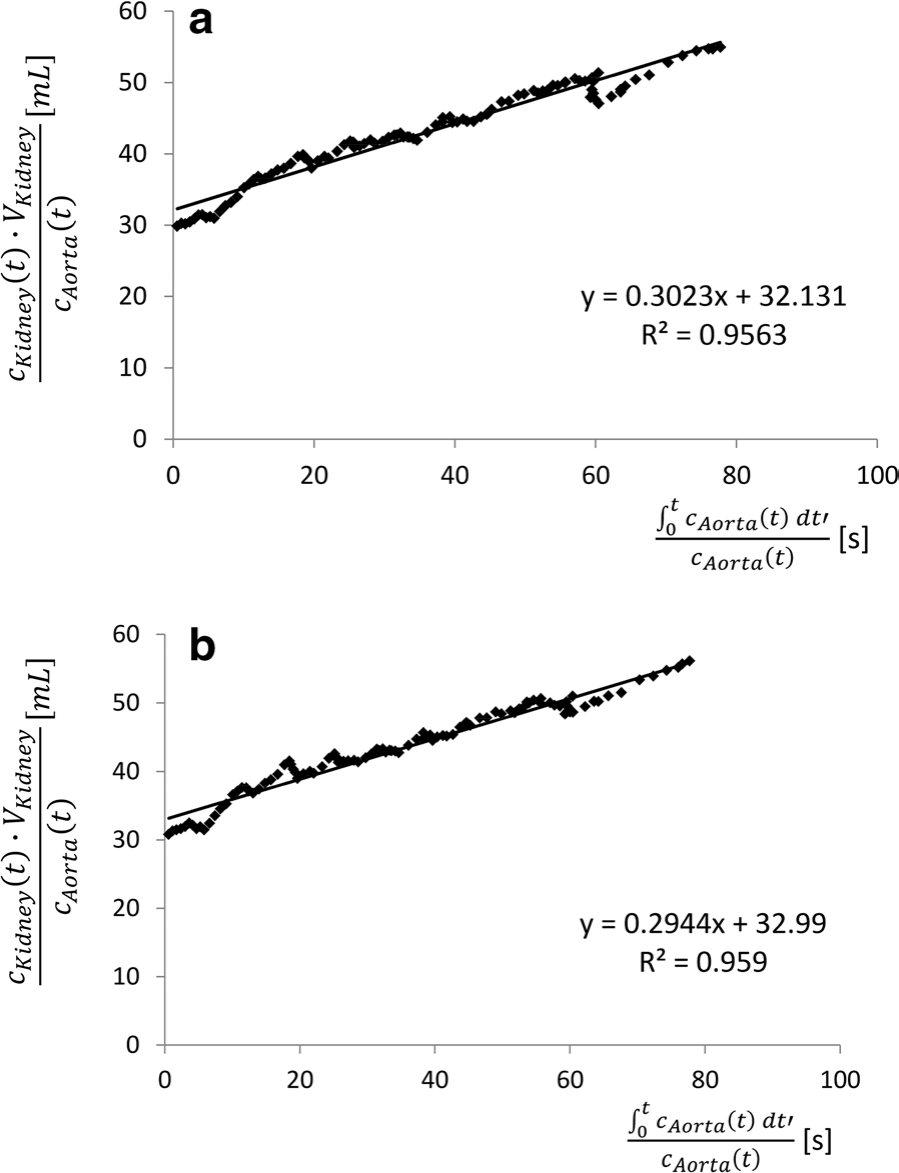
Exemplary RPP (evaluation interval only) of one dog. **a** The RPP of the left kidney and **b** the RPP of the right kidney. The dots display the measured values of the RPPs and the black line the trend line of the RPP. R^2^ = 0.96 indicates a good fit of the regression line

For all eight dogs, the renal clearance of contrast medium was calculated using an RPP. The mean value of Δt, which is the time span for shifting the AIF, was 6.1 ± 0.8 s. The results of both kidneys of one dog are displayed in Fig. 9. The gradient of the trend line represents the renal clearance of the contrast medium.

**Fig. 10:**
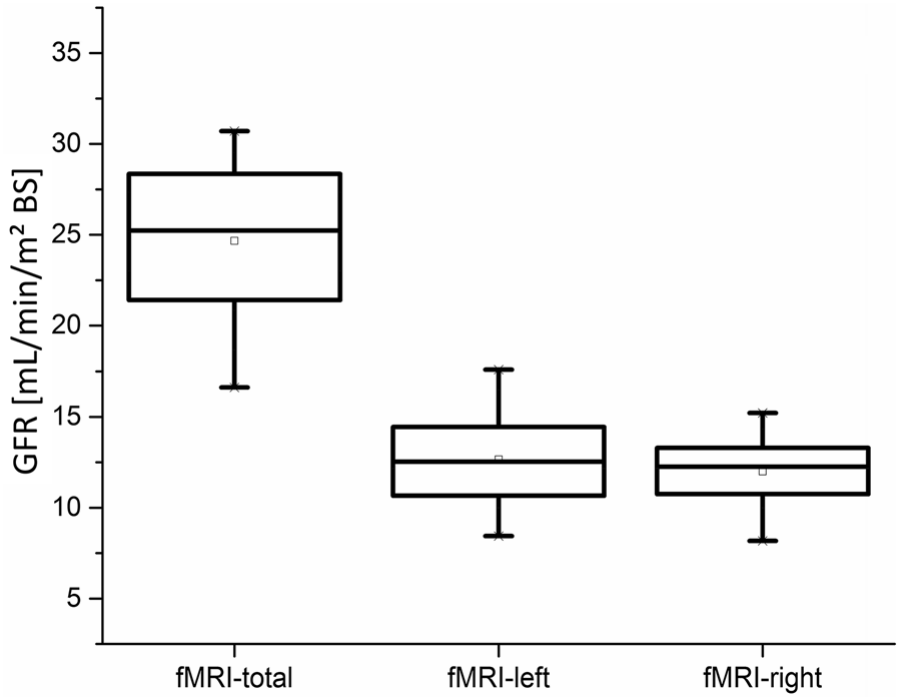
Results of the GFR-calculation based on RPPs. The complete GFR was calculated by summarising the single-kidneys GFRs. Comparisons between the left and the right kidney resulted in pretty similar results

A summarised presentation of all results can be seen in Fig. 10. The total GFR of our measurements amounted to an average of 24.7 ± 4.8 mL/min/m^2^ BS. The mean value of the left kidney was 12.7 ± 2.9 mL/min/m^2^ BS and on the right side 12.0 ± 2.2 mL/min/m^2^ BS. R^2^-values of the regression lines were on average 0.91 ± 0.08.

**Fig. 11:**
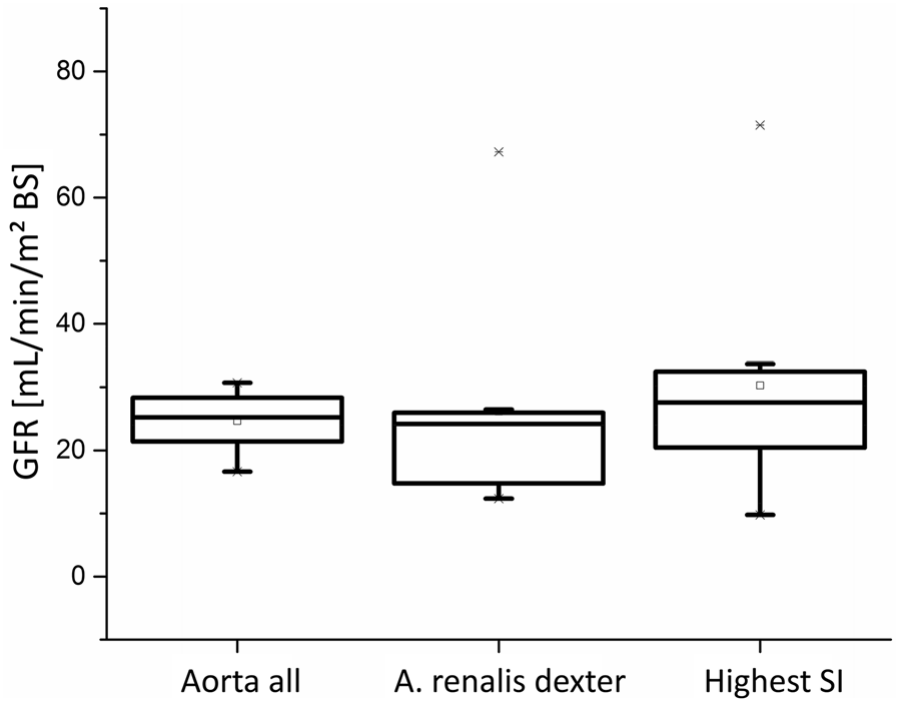
Influence of varying localisations of ROI_aorta_ on the GFR-calculations via RPP. The different places of the ROIs can be seen in Fig. 3. The total-GFR is displayed in order to visualise the impact of the size and localisation of ROI_aorta_

Another aspect of analysing the results of the RPPs was the choice of the size and localisation of ROI_aorta_. The localisations of the different ROIs_aorta_ can be seen in Fig. 1. The results are displayed in box-plots in Fig. 11. The localisation of ROI_aorta_ for determining the area under the curve (AUC) had a great influence on the calculated results of the RPP.

The mean values ranged from 24.7 ± 4.8 mL/min/m^2^ BS (ROI_aorta all_) to 26.2 ± 17.6 mL/min/m^2^ BS (ROI_a.renalis dexter_) to 30.3 ± 18.3 mL/min/m^2^BS (ROI_highest SI_). The size of the ROI had an effect on the results, too. With an increasing size of the ROIs the spread of the results was minimised.

## Discussion

### Bolus propagation

For all eight dogs, a timely similar but individually diverse progression of contrast medium could be monitored according to the different ROIs: The time gap of 7.0 s for reaching the first maximum in ROI_aorta_ and the differences for proceedings to the further extremes underline the varying propagation of the bolus in the different dogs. Obviously, if the first maximum was reached early, also the other extreme values were reached early in comparison to the other dogs.

For the calculated concentrations similar observations were found with an even higher dispersion of the results. On the one hand, these grave fluctuations of the bolus propagation in the different dogs could be explained by the fact that the bolus was administered manually. This kind of administering is never as exact as an automatic administration. On the other hand, variations in blood pressure and depth of anaesthesia should be considered as influencing factors as well as the body weight.

As demonstrated in Figs. 7 and 8 hardly any differences could be monitored for the comparisons between the left and right kidney of each dog concerning extreme values of time or concentrations. When comparing the ROIs_cortex_ to ROIs_kidney_ also just marginal differences were detected. This aspect emphasises that the comparison of single kidney function with this method should give a clue on lower kidney function in comparison to the other side.

### Rutland-Patlak Plot

As demonstrated in previous studies there is no final consensus concerning the evaluation interval, which should be used for calculating the slope of the RPP [19, 29]. In this study, the ideal evaluation interval in the RPPs should be identified. In order to fulfil all requirements of the RPP-model, the delayed increase in the contrast medium in the kidneys in comparison to the aorta has to be considered.

Due to the different propagation of the contrast medium bolus in the dogs, the identification of the ideal evaluation interval for the RPPs was difficult. For about the first 30 s after the arrival of the contrast medium in the kidneys the perfusion of the renal vessels has the greatest influence on the measurements of the SIs. To rule out this influence, the best starting point for integration was identified at that point in time when the second maximum of the Gd-DOTA concentration in the aorta could be measured. For all dogs, an ascending regression line could be measured after this point in time in the RPP. This regression line reveals the clearance of the contrast medium.

The end of the evaluation interval was set after 60 s. After about 70 s for some dogs a decrease in data values in the RPPs occurred. This might be caused by the elimination of the contrast medium into the urinary bladder. At this point in time not all pre-conditions of the RPP-model would be fulfilled.

The evaluation interval of the RPP was set 60 s after the second maximum in the aorta as more than 100 data points were included for the calculation and the R^2^ values (R^2^ = 0.91 ± 0.08) of the regression lines of the RPPs indicate a good model fit for so many data points. This time interval fits well to the results described for ^99m^Tc-DTPA in scintigraphy. They found the best time interval for integration to be between 30 and 120 s [30].

As presented in Fig. 10 the comparison between left and right kidney function resulted in pretty similar outcomes. For all other dogs the difference never exceeded 5 mL/min/m^2^ BS and was statistically insignificant (p = 0.65). This corresponded to our expected results of the healthy dogs.

As described above a so-called aortic input function (AIF) is needed to determine the concentration of contrast medium in the vascular space. Besides the susceptibility of blood vessels to artefacts in MRI measurements [17] there is a great impact of the choice of ROI_aorta_ on the computed concentrations [31]. Even in human medicine there is no standard process for calculating the AIF to enable an interindividual comparison [32].

The demonstrated results in Fig. 11 emphasise these aspects in our study, too. This leads to a need for standardised choice of ROIs for comparisons between single patients and different compartment models.

In this study, the results obtaining the lowest standard deviation were found when ROI_aorta_ was selected as large as possible.

Comparisons between the GFR results of our study and the creatinine clearance method are difficult to be performed. In our study, the results from measuring the GFR via fMRI had much lower values than those from measuring the creatinine clearance.

A main point is the impact of the anaesthesia on the GFR measurements via fMRI-examinations. Lower results for GFR-measurement have been detected for patients under anaesthesia than without [33, 34]. Additionally, the results for the GFR are dependent on the method used for the measurement and the contrast medium.

Another difference between the creatinine clearance method and the fMRI measurements was the period of time in which GFR was calculated. The time interval for estimating GFR via exogenous plasma creatinine clearance was at least 4 h, whereas the period of time for measuring GFR via MRI bolus track was 60 s. Additionally, the single kidney function could only be measured by the fMRI measurements and not by a blood sampling strategy.

### Image analysis

One challenge of the MRI bolus track evaluation was to eliminate the artefacts caused mainly by breathing movement. Two different approaches could be selected. It is possible to reduce breathing movements during examination by deep anaesthesia and manual breathing triggering. Another approach would be to eliminate these artefacts during post-editing of the images. Instead of manual corrections [18], a Savitzky–Golay filter [26] was used. As demonstrated in Fig. 5 the use of the filter showed good approximations concerning curve propagation and extreme values. This filter presented itself as a simple and non-time-consuming method for eliminating these artefacts without losing information about curve propagation or extreme values. The use of the filter also simplified the identification of the “point after aortic rise” because for some dogs breathing movement occurred at the same time as the propagation of the contrast medium bolus started.

The slice thickness of 45 mm had some advantages and disadvantages. A positive aspect of this approach was that changes of SI could be measured for the whole kidney. Furthermore, both kidneys and the aorta were completely captured in one slice so that the calculation was not limited to a small proportion of the renal parenchyma [18] and the contrast medium remained completely in the ROIs during the time interval of analysis [35]. Further possible error sources might occur due to the usage of the additional ROIs that had to be established for calculations of the correction factors. As the slice thickness has a wider range than the volume of the organs the residual tissue has to be subtracted. This might lead to some falsifications because of the possible localisations of these ROIs especially for ROI_aorta_.

General disadvantages of this method are the great influence of the size and localisation of the ROIs on the calculations and the possible nephrotoxicity of the contrast medium [36] especially if repeated examinations are scheduled. A further general weakness is the lack of validated single kidney GFR reference values for dogs and the non-existence of published data about a clinically relevant change in fMRI-GFR measurements [37]. This complicated the interpretation of the calculated results extremely.

The choice of the sectional plane affects the results, too. If a coronal sectional plane is chosen for MRI measurement the options of ROI selection are more restricted due to anatomical facts. In our opinion the definition of ROI selections is one of the key issues to establish a clinical routine measurement method as there are a lot of differences according to breed in the dog population. This might cause some challenges in defining standardised ROIs.

Another advantage is the short duration of this method compared to different blood sample methods.

## Conclusions

The propagation of the contrast medium bolus could be depicted well. The propagation of the contrast bolus proceeded in a similar manner for every individual dog, too. Additionally, the comparison of the single kidney function of the individual dogs is possible with this method.

A standardised examination procedure (anaesthesia-protocol, administering the contrast medium automatically, localisations and size of the ROIs and determining the time interval for the integration of the RPPs) would be recommended in order to minimise influencing parameters.

As the ideal vision of the kidney examination would be a procedure in which the kidneys could be judged morphologically and functionally in one examination, MRI examinations of the kidneys seem to be a promising tool to solve this problem.

## Additional file

**Additional file 1.** An additional time-lapse movie file shows the time-dependent distribution of the contrast medium in a dog.
